# Acquired resistance to AZD9291 as an upfront treatment is dependent on ERK signaling in a preclinical model

**DOI:** 10.1371/journal.pone.0194730

**Published:** 2018-04-11

**Authors:** Bo Mi Ku, Moon Ki Choi, Jong-Mu Sun, Se-Hoon Lee, Jin Seok Ahn, Keunchil Park, Myung-Ju Ahn

**Affiliations:** 1 Samsung Biomedical Research Institute, Samsung Medical Center, Sungkyunkwan University School of Medicine, Seoul, Korea; 2 Center for Colorectal Cancer, Research Institute and Hospital, National Cancer Center, Goyang, Korea; 3 Division of Hematology-Oncology, Department of Medicine, Samsung Medical Center, Sungkyunkwan University School of Medicine, Seoul, Korea; Seoul National University College of Pharmacy, REPUBLIC OF KOREA

## Abstract

AZD9291 (osimertinib) is approved for standard care in patients with EGFR T790M-positive non-small cell lung cancer (NSCLC) after prior EGFR TKI progression. Furthermore, AZD9291 is now being evaluated as a first-line treatment for NSCLC patients with activation EGFR mutations. Based on previous experiments, resistance to AZD9291 as a first-line treatment may also emerge. Thus, identification and understanding of resistance mechanisms to AZD9291 as a first-line treatment can help direct development of future therapies. AZD9291-resistant cells (PC9/AZDR) were established using EGFR inhibitor-naïve PC9 cells. Resistance mechanisms were analyzed using next-generation sequencing (NGS) and a proteome profiler array. Resistance to AZD9291 developed through aberrant activation of ERK signaling by an EGFR-independent mechanism. The combination of a MEK inhibitor with AZD9291 restored the sensitivity of PC9/AZDR cells *in vitro* and *in vivo*. PC9/AZDR cells also showed increased MET expression and an HRAS G13R mutation. In addition, maspin expression was higher after AZD9291 treatment in PC9/AZDR cells. Sustained ERK activation confers resistance to AZD9291 as a first-line therapy. Thus, co-targeting EGFR and MEK may be an effective strategy to overcome resistance to AZD9291.

## Introduction

Targeted therapy using the first-generation EGFR tyrosine kinase inhibitors (TKIs) gefitinib/erlotinib and second-generation afatinib is substantially better than standard chemotherapy in patients with non-small cell lung cancer (NSCLC) harboring activating EGFR mutations [1]. However, almost all patients exhibit acquired resistance to EGFR TKIs and will ultimately experience relapse within one year. Several mechanisms, including secondary EGFR mutations, activation of bypass signaling, and histologic transformation, have been identified for acquired resistance to EGFR TKIs. The major cause of resistance is the secondary EGFR T790M gatekeeper mutation (50%~60%) [1, 2].

Third-generation irreversible EGFR TKIs (AZD9291 and CO1686) specifically target EGFR T790M as well as the activating EGFR mutations, but spare wild-type EGFR. In a phase I clinical trial, AZD9291 (osimertinib) and CO1686 (rociletinib) have shown promising clinical activity in patients with EGFR T790M-mediated acquired resistance to first- or second-generation EGFR TKIs with response rates of 61% and 59%, respectively [3, 4]. Based on recent phase II and phase III trials, AZD9291 represents the best option in the acquired resistance setting [5, 6].

However, despite impressive responses in EGFR T790M-positive patients, acquired resistance to AZD9291 eventually occurs. The main mechanism of resistance to AZD9291 is the EGFR C797S mutation in the kinase-binding site, which accounts for 33–36% of AZD9291-treated patients [7, 8]. In addition, activation of the MAPK pathway and emergence of amplified MET have been found as drivers of resistance to AZD9291 [9–11].

Although AZD9291 has been approved as second-line therapy in patients with EGFR-TKI pre-treated, EGFR T790M-positive, advanced NSCLC, AZD9291 is now being tested as a first-line treatment for NSCLC patients with activating EGFR mutations. A randomized phase III trial comparing osimertinib with gefitinib or erlotinib (FLAURA) demonstrated that osimertinib significantly improved progression-free survival (18.9 months *vs* 10.2 months, HR 0.46) [12]. Because previous experience with EGFR TKIs suggests that resistance to AZD9291 as a first-line therapy may also emerge and potentially limit its therapeutic effect, identification of resistance mechanisms is crucial to guide further treatment.

Therefore, we evaluated mechanisms of acquired resistance to AZD9291 as a first-line therapy in TKI-naïve NSCLC harboring activating EGFR mutations. We found that EGFR-independent activation of ERK is a critical event that mediates resistance to AZD9291 as a first-line therapy for TKI-naïve NSCLC.

## Materials and methods

### Chemical reagents and antibodies

AZD9291 and AZD6244 were provided by AstraZeneca Pharmaceuticals. Erlotinib, gefitinib, afatinib, CO1686, crizotinib, capmatinib, cabozantinib, MGCD-265, and merestinib were purchased from Selleckchem. All drugs were dissolved in dimethyl sulfoxide (DMSO) at a 10 mM concentration and stored in small aliquots at -20°C until further use. Antibodies specific for p-EGFR (Tyr1068), EGFR, p-AkT (Ser473), AkT, p-ERK1/2 (Thr202/Tyr204), ERK1/2, p-MET (Tyr1234/1235), MET, and Ki-67 were obtained from Cell Signaling Technologies. HRAS, Maspin, and β-actin antibodies were obtained from Santa Cruz Biotechnology. HRAS siRNA, Maspin siRNA and control siRNA were purchased from Santa Cruz Biotechnology.

### Cell lines and transfection

PC9 cells were obtained from the ATCC and cultured in RPMI-1640 medium supplemented with 10% FBS, penicillin (100 U/ml), and streptomycin (100 μg/ml) at 37°C in a humidified atmosphere containing 5% CO_2_. Cell line identity was authenticated by short tandem repeat analysis. The AZD9291-resistant cell line PC9/AZDR was newly established in our laboratory by exposing PC9 cells to gradually increasing concentrations of AZD9291 (starting at 10 nM and ending with 1 μM) for approximately 6 months. The established cells maintained resistance to AZD9291 even after withdrawal of AZD9291 from the culture medium. Transient siRNA transfections of PC9 or PC9/AZDR cells were performed using RNAiMAX (Invitrogen) according to the manufacturer’s protocol. Cells were harvested 48 h after transfection.

### Cell viability and proliferation assay

Cells were seeded on a 96-well plate, allowed to adhere overnight, and treated with the indicated drugs for 72 h. Cell viability was determined using a Cell Counting Kit (Dojindo Molecular Technologies) according to the manufacturer’s instructions. IC_50_ values were calculated using nonlinear regression analysis of GraphPad Prism. Cell proliferation rate at 48 h after treatment was measured with BrdU cell proliferation assay Kit (Cell signaling Technologies) according to the manufacturer’s instructions.

### Genetic analysis

EGFR mutations were evaluated by PCR, followed by sequencing reactions with Sanger sequencing chemistry using a BigDye^®^ Terminator v3.1 Cycle Sequencing kit (Applied Biosystems Inc., Foster City, CA, USA) on an ABI 3730XL automated sequencer (Applied Biosystems, USA). Amplicon-based targeted next-generation sequencing (NGS) analysis was performed using a customized cancer panel (CancerSCAN^™^) designed to enrich exons of 83 genes.

### Western blot analysis

Cells were lysed on ice in NP-40 lysis buffer supplemented with a protease and phosphatase inhibitor cocktail (Sigma). Equal amounts of protein were then subjected to SDS-PAGE and transferred to polyvinylidene difluoride (PVDF) membranes. After blocking, membranes were incubated overnight at 4°C with the indicated antibodies and developed by ECL.

### Proteome profiler array

The Human XL Oncology Array Kit (R&D Systems) was used for the parallel determination of relative levels of 84 human cancer-related proteins. The Human Phospho-kinase Array Kit (R&D Systems) was used to measure relative levels of phosphorylation of 43 kinase phosphorylation sites. Cell lines were treated with AZD9291 for 24 h and arrays were done according to the manufacturer’s protocol.

### Xenograft studies

The protocol involving all procedures about animals was reviewed and approved by the Institutional Animal Care and Use Committee (IACUC) at Samsung Biomedical Research Institute (SBRI) (Permit Number:20160113001). They are in accordance with the relevant national and international guidelines. Mice were obtained from Orient Bio Inc. (Seongnam, Korea) and housed 5 per cage in ventilated cages with free access to food and water. Six-week-old BALB/c female nude mice were injected subcutaneously with PC9/AZDR cells. When tumor size reached approximately 200 mm^3^, mice were randomly assigned to groups of 9–10 mice each. Each group of mice was dosed by daily oral gavage with vehicle, AZD9291 (5 mg/kg/d), or AZD6244 (10 mg/kg/d). AZD9291 and AZD6244 were dissolved in 1% Tween-80 and 0.5% hydroxypropyl methylcellulose plus 0.1% Tween-80, respectively. Tumor volumes were determined using calipers and calculated using the following formula: V = (L x W^2^)/2 (L, Length; W, width) and the tumors were removed for immunohistochemistry. Mice were monitored daily with humane endpoints including a tumor greater than 1500 mm^3^, a weight loss of over 15% of body mass, vomiting or skin problems, or inability to ambulate or rise for food and water. These humane endpoints were not observed in any mouse. All efforts were made to alleviate suffering. Mice were euthanized by CO_2_ inhalation at the end of experiment.

### TUNEL and immunohistochemistry

For terminal deoxynucleotidyl transferase-mediated dUTP nick end labeling (TUNEL) staining, cells were cultured on coverslips and treated with indicated drugs for 48 h. TUNEL staining was done by In situ Apoptosis Detection Kit (Takara) according to the manufacturer’s instruction. Cell nuclei were counterstained with 4’,6-diamidino-2-phenylindole (DAPI). Hematoxylin and eosin (H&E) and Ki-67 staining were performed on formalin-fixed, paraffin-embedded tissues from mice xenografts.

### Statistical analysis

Data are presented as the mean ± SE. Statistical analyses were carried out using GraphPad Prism (GraphPad software). A *p* value <0.05 was considered statistically significant.

## Results

### Characteristics of AZD9291-resistance after first-line therapy

**Fig 1:**
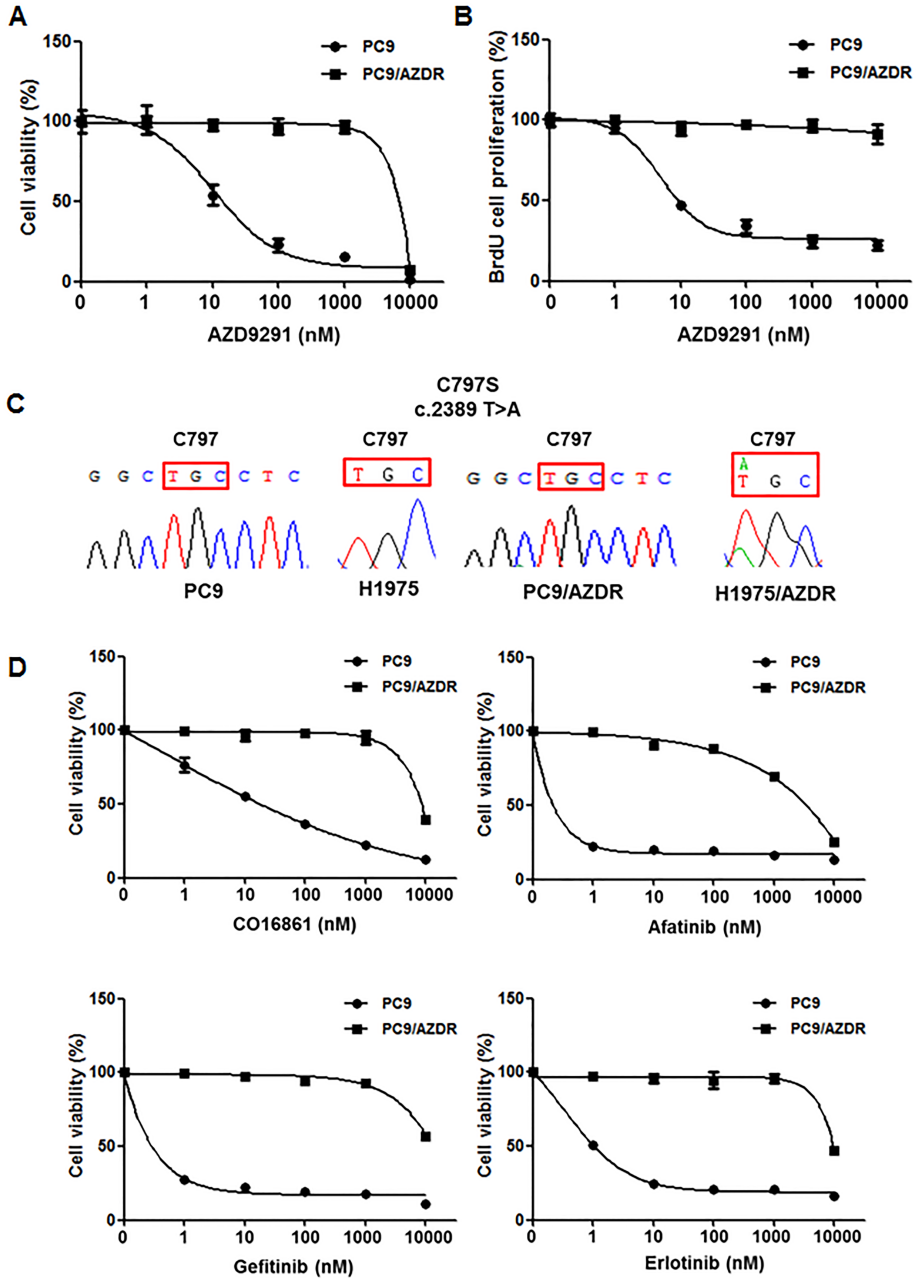
Acquired resistance to AZD9291 as first-line treatment is mediated by EGFR-independent mechanisms. (A) PC9 and PC9/AZDR cells were treated with the indicated concentrations of AZD1775 for 72 h. Cell viability was determined using CCK-8 assay. The data are mean ± SE of six replicates. (B) Cell proliferation was measured by BrdU cell incorporation at 48 h after treatment. The data are mean ± SE of six replicates. (C) Sanger sequencing of EGFR cDNA clones derived from PC9/AZDR. (D) PC9 and PC9/AZDR cells were treated with the indicated concentrations of CO-1686, afatinib, gefitinib, erlotinib for 72 h. Cell viability was determined using the CCK-8 assay. The data are mean ± SE of six replicates.

To demonstrate the resistance mechanism of AZD9291 as a first-line therapy in an EGFR T790M-negative setting, we established AZD9291-resistant PC9 cells (PC9/AZDR) using a dose-escalation method. This cell line showed resistance to AZD9291 in drug concentrations >1000-fold (1μM) the initial IC_50_ of the parental PC9 cells (Fig 1A and 1B). PC9/AZDR cells were also resistant to other EGFR TKIs such as CO1686, afatinib, gefitinib, and erlotinib (Fig 1C). As the EGFR C797S mutation was the most common resistance mechanism to AZD9291 in T790M-positive NSCLC patients who failed prior EGFR TKIs [8, 13], we analyzed this mutation in PC9/AZDR cells. In contrast to EGFR T790M-expressing H1975 cells, mutation analysis of *EGFR* by direct sequencing found no acquired EGFR C797S mutation in PC9/AZDR cells (Fig 1B), suggesting that a bypass signaling mechanism may be activated. Concordant with previous studies [9, 10], EGFR T790M has not emerged as a resistance mechanism to AZD9291 (S1 Fig).

### ERK signaling mediates resistance to AZD9291 in PC9/AZDR cells

**Fig 2:**
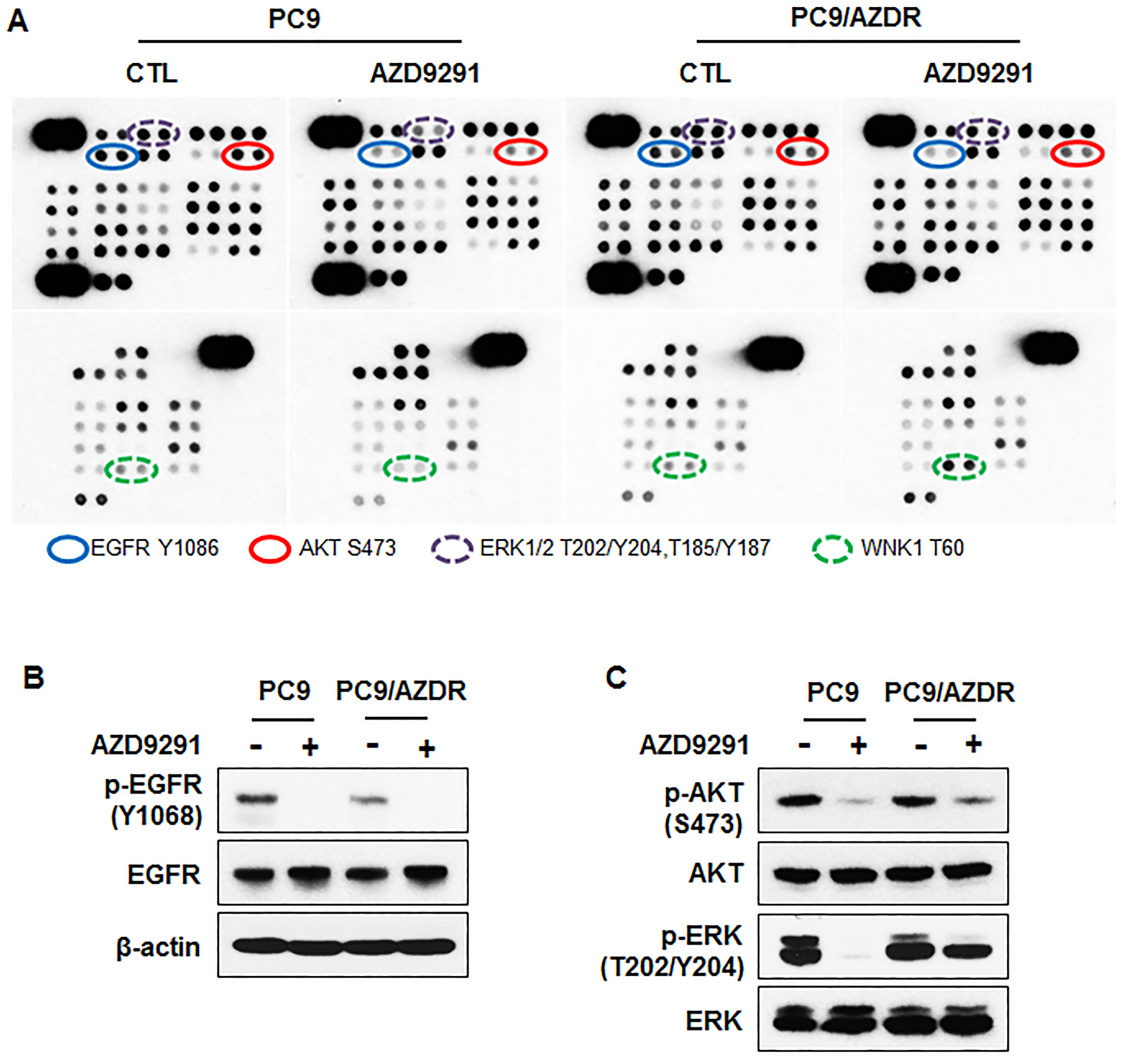
AZD9291-resistant PC9 cells exhibit persistent ERK activation. (A) Relative levels of phosphorylation of 43 kinase phosphorylation sites were compared between PC9 and PC9/AZDR cells after 24 h treatment with 100 nM AZD9291. (B) Activation of EGFR and (C) its downstream signaling AKT and ERK were confirmed by Western blotting.

To determine the role of alternative signaling pathways in resistance, 43 different kinase phosphorylation patterns were analyzed using a phospho-kinase array in the presence and absence of AZD9291. The phospho-kinase array showed inhibition of EGFR phosphorylation but sustained phosphorylation of ERK in AZD9291-treated PC9/AZDR cells (Fig 2A). In addition, WNK1 phosphorylation only increased in resistant cells after AZD9291 treatment. These changes of p-EGFR and p-ERK were confirmed by Western blotting. AZD9291 still inhibited EGFR phosphorylation in the resistant cells, although basal EGFR phosphorylation was slightly lower in resistant cells compared to parental cells (Fig 2B). However, ERK phosphorylation was maintained after AZD9291 treatment despite inhibition of EGFR signaling (Fig 2C). The discrepancy between EGFR and ERK phosphorylation suggests EGFR-independent mechanisms of resistance to first-line AZD9291 in PC9/AZDR cells.

**Fig 3:**
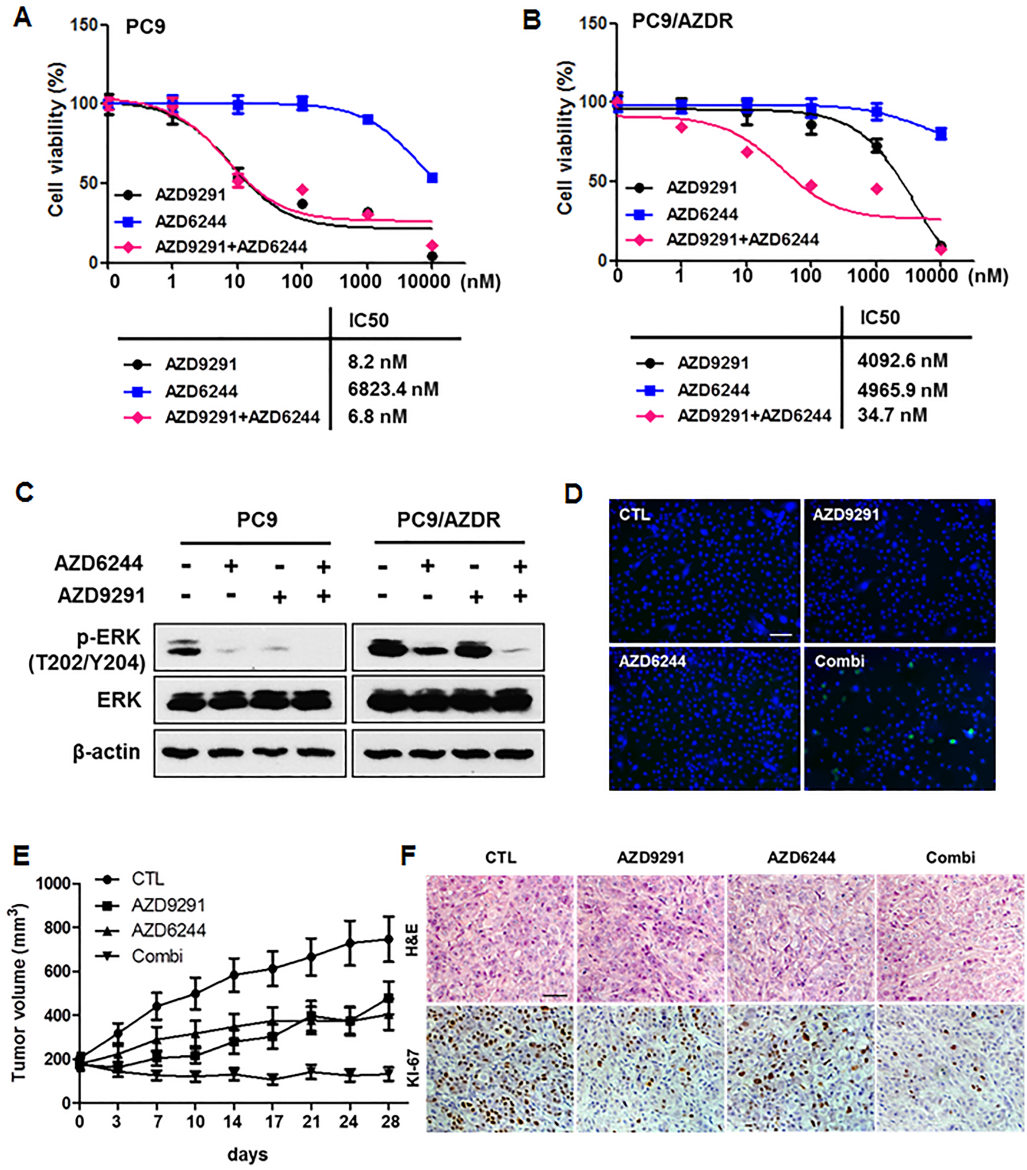
The combination of AZD9291 with a MEK inhibitor effectively inhibits the growth of AZD9291-resistant cells. (A) PC9 and (B) PC9/AZDR cells were treated with various concentrations of AZD9291 alone, AZD6244 alone, or their combinations for 72 h. The data are mean ± SE of six replicates. (C) Cells were treated with 100 nM AZD9291 alone, or 100 nM AZD6244 alone, and the combination of AZD9291 with AZD6244 for 24 h. Western blotting was carried out to determine the levels of p-ERK. β-actin was used as a loading control. (D) TUNEL staining was performed after 48 h treatment of AZD9291 (100 nM), AZD6244 (100 nM), and the combination of AZD9291 with AZD6244. Nuclei were counterstained with DAPI. Scale bar, 50 μm. (E) PC9/AZDR xenografts were treated with vehicle, AZD9291 (5 mg/kg/d), AZD6244 (10 mg/kg/d), or AZD9291 plus AZD6244 by oral gavage for 5 days each week for a total of 4 weeks. Tumor sizes were measured as indicated. Each measurement is mean ± SE of 9–10 replicates. (F) Xenograft tumor sections were stained with H&E and Ki-67. Nuclei were counterstained with hematoxylin in Ki-67 stained slides. Scale bar, 100 μm.

To demonstrate the role of ERK signaling in inducing resistance in PC9/AZDR cells, we tested the anti-tumor effects of combined treatment with AZD9291 and the MEK inhibitor, AZD6244. This combination was more synergistic than either single agent in PC9/AZDR cells (Fig 3B), but not in PC9 parental cells (Fig 3A). Consistent with this, AZD6244 treatment in the presence of AZD9291 resulted in complete inhibition of ERK phosphorylation (Fig 3C) and induced apoptosis (Fig 3D). Following *in vitro* combination studies, we then examined the efficacy of combined AZD9291 and AZD6244 against the growth of PC9/AZDR tumors *in vivo*. Although tumor growth inhibition occurred with AZD9291 or AZD6244 alone, the combination more significantly inhibited tumor growth than either single agent (Fig 3E), as well as significantly reduced tumor cell proliferation (Fig 3F). The combinations were deemed tolerable, because no significant body weight reduction was observed during the treatment period (S2 Fig). Though inhibition of ERK signaling by a MEK inhibitor increased sensitivity to AZD9291 in PC9/AZDR cells, AZD9291 resistance was not completely overcome.

### Identification of an HRAS G13R mutation in AZD9291 resistant cells

**Fig 4:**
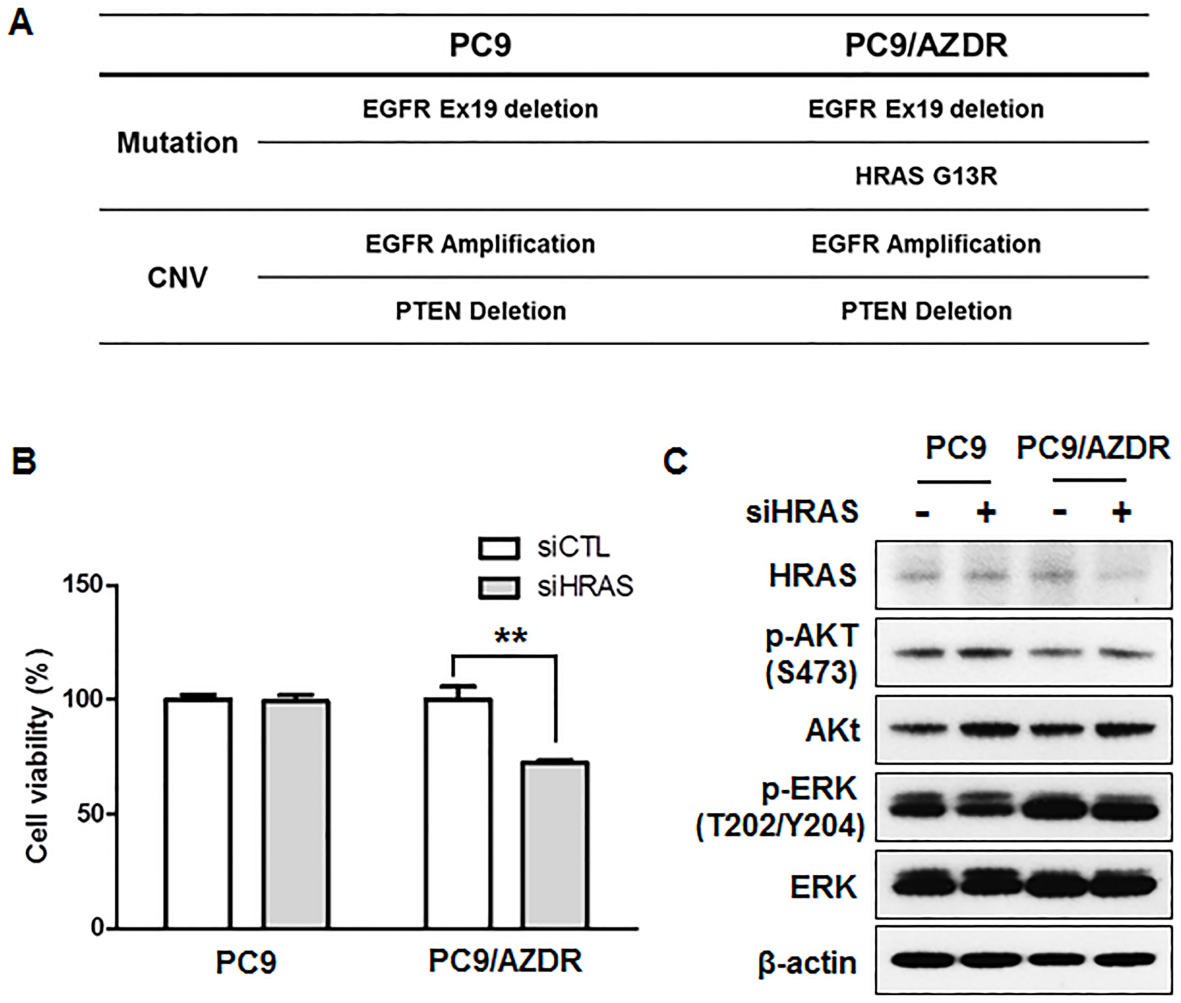
AZD9291-resistant PC9 cells contain an HRAS mutation. (A) Summary of genetic alterations in PC9 and PC9/AZDR cells. (B) Cell viability was measured by CCK-8 assay of cells transfected with HRAS siRNA for 48 h. The data are mean ± SE of six replicates. **, *P* < 0.01 for comparison of the indicated pairs. C. Cells were transfected with either nontargeting control siRNA (siCTL) or HRAS siRNA (siHRAS) for 48 h. HRAS knockdown and phosphorylation of AKT and ERK were detected by Western blotting. β-actin was used as a loading control.

To further investigate the resistance mechanism, we conducted a targeted NGS analysis of PC9/AZDR cells and compared them to the parental PC9 cells. The NGS panel contains gene mutations, fusion, and copy-number variations across 375 cancer-related genes. The PC9/AZDR cells contained an HRAS G13R mutation which was not present in the parental drug-sensitive cell line (Fig 4A). To determine whether the HRAS G13R mutation was required for AZD9291 resistance, HRAS expression was knocked down by siRNA in PC9 and PC9/AZDR cells, which led to a partial reduction of cell viability in PC9/AZDR cells as compared with PC9 parental cells (Fig 4B). However, HRAS inhibition had no effect on ERK phosphorylation in PC9/AZDR cells (Fig 4C), indicating involvement of other receptor tyrosine kinases for ERK activation.

### Comparison of cancer-related protein expression

**Fig 5:**
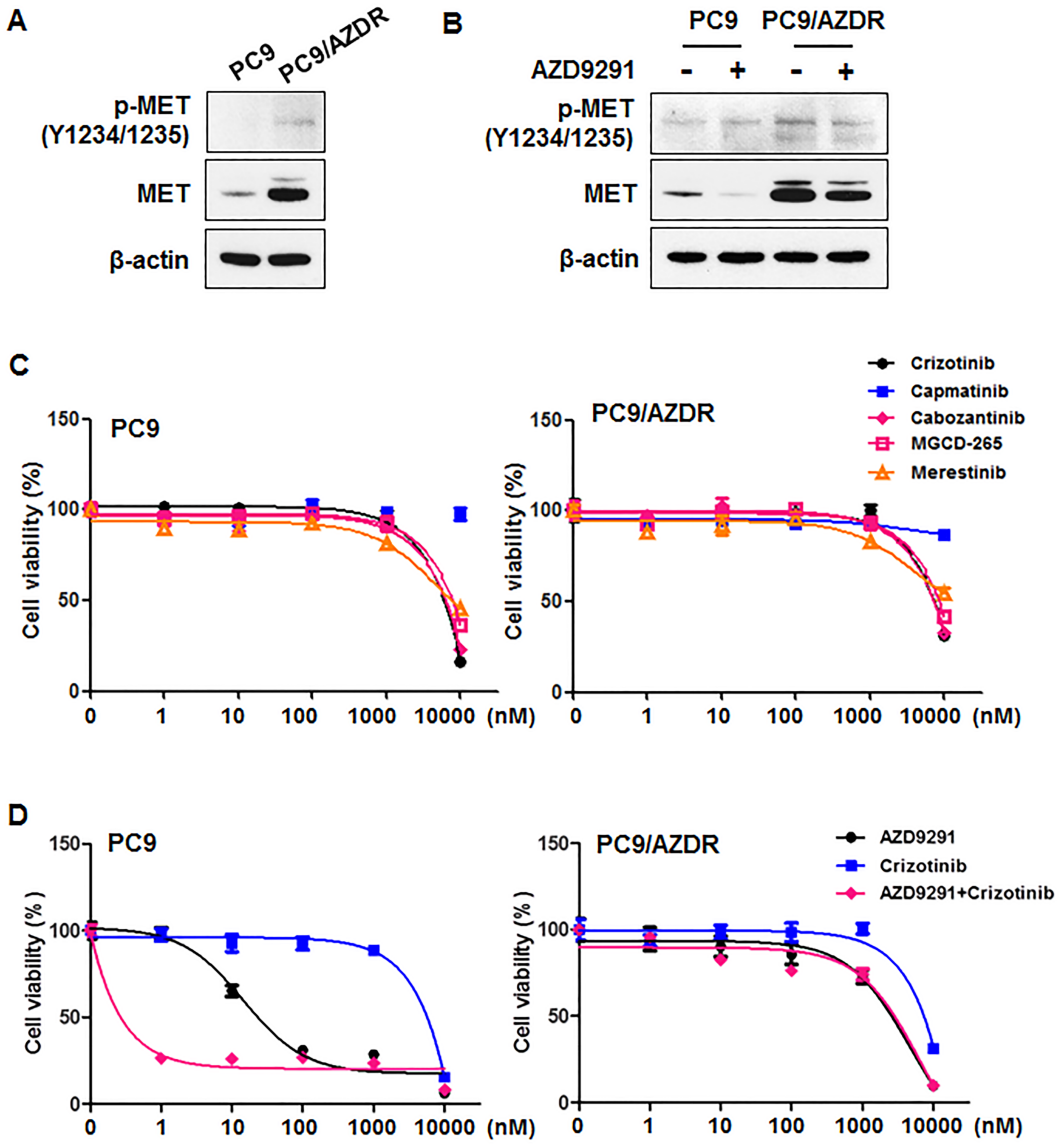
AZD9291-resistant PC9 cells show increased MET expression levels. (A) Cell lysates obtained from PC9 and PC9/AZDR were analyzed by Western blotting using the indicated antibodies. (B) Relative expression levels of p-MET and MET were compared between PC9 and PC9/AZDR cells after 24 h treatment of 100 nM AZD9291. β-actin was used as a loading control. (C) PC9 (left) and PC9/AZDR (right) cells were treated with various concentrations of MET inhibitors, crizotinib, capmatinib, cabozantinib, MGCD-265, and merestinib, for 72 h. The data are mean ± SE of six replicates. (D) PC9 (left) and PC9/AZDR (right) cells were treated with various concentrations of AZD9291 alone, crizotinib alone, or their combinations for 72 h. The data are mean ± SE of six replicates.

MET amplification is a well-known bypass mechanism of EGFR TKI resistance [1, 11, 14], MET amplification was not detected in the NGS analysis of PC9/AZDR cells. However, MET expression was considerably higher in PC9/AZDR cells relative to baseline in PC9 cells (Fig 5A). In contrast to a substantial increase of MET expression, MET phosphorylation was hardly changed in resistant cells compared with parental cells. In addition, MET expression was lower in PC9 cells, but not in PC9/AZDR cells, after AZD9291 treatment (Fig 5B). These results suggest that AZD9291 may induce MET degradation in drug-sensitive conditions, increasing MET expression in AZD9291-resistant cells regardless of MET amplification. To determine whether increased MET expression was crucial in AZD9291-resistance cells, we treated cells with five different MET inhibitors and evaluated cell viability. Despite increased MET expression, the MET inhibitors had no effect on cell viability in PC9/AZDR cells (Fig 5C). In addition, the combination of AZD9291 and crizotinib had no synergistic effects in PC9/AZDR cells (Fig 5D), and other MET inhibitors showed similar effects (S3 Fig). These results indicate that increased MET expression was not the cause of AZD9291 resistance, but rather the result of resistance.

**Fig 6:**
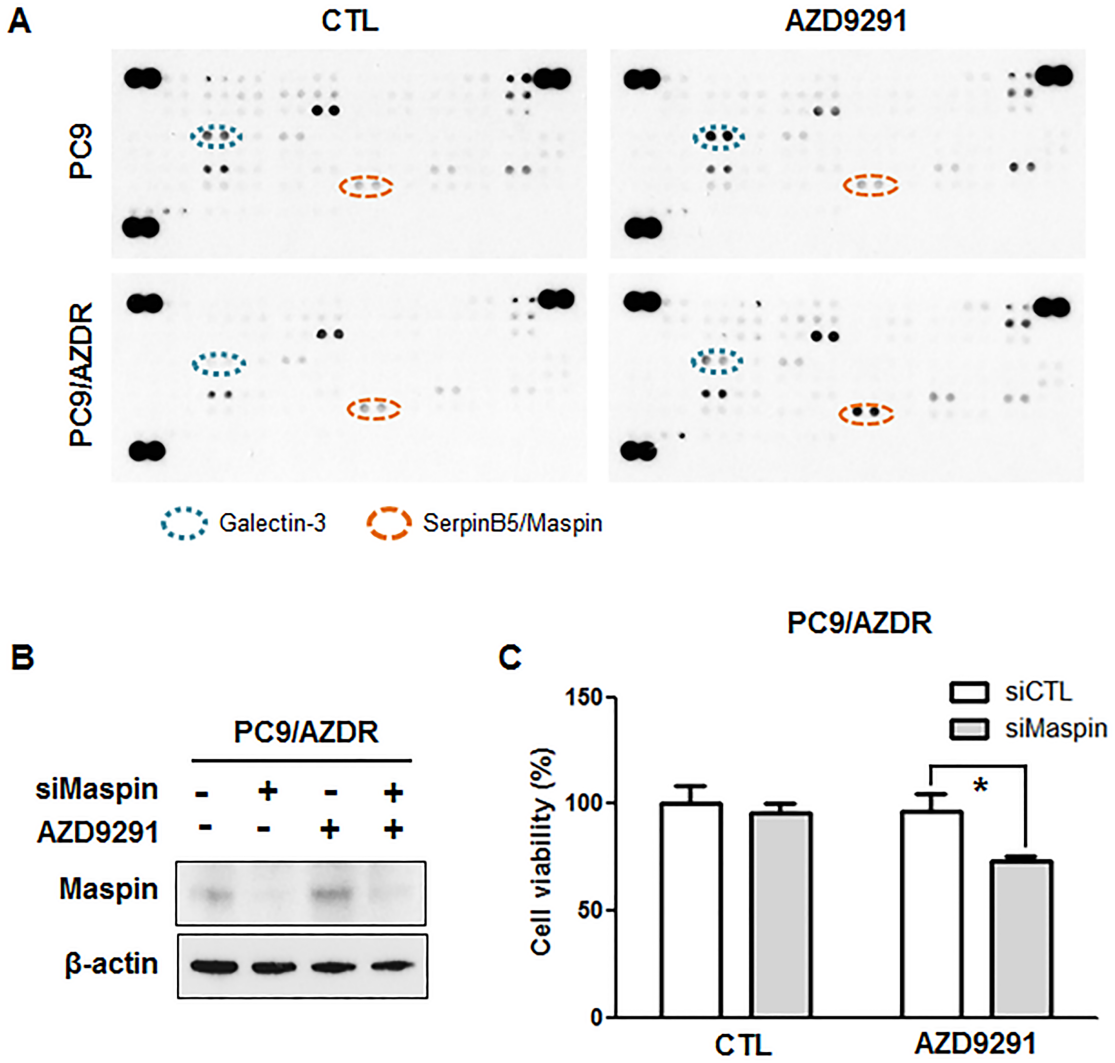
AZD9291-resistant PC9 cells show increased SerpinB5 levels after AZD9291 treatment. (A) A Human XL Oncology Array was used to determine candidates for resistance-related proteins by comparison of PC9 and PC9/AZDR cell lysate after 24 h treatment with 100 nM AZD9291. (B) Cells were transfected with either nontargeting control siRNA (siCTL) or maspin siRNA (siMaspin) for 24 h and treated with 100 nM AZD9291 for 24 h. Maspin knockdown was confirmed by Western blotting. β-actin was used as a loading control. (C) After 24 h after siRNA transfection, cells were treated with 100 nM AZD9291 for 48 h. Cell viability was measured by CCK-8 assay. The data are mean ± SE of six replicates. *, *P* < 0.05 for comparison of the indicated pairs.

To evaluate more cancer-related protein expression changes, we utilized a proteome profiler array. Expression of 84 cancer-related proteins was determined after 24 h of AZD9291 treatment. After AZD9291 treatment, galectin-3 expression increased in PC9 parental cells, but not in PC9/AZDR cells (Fig 6A). Among the 84 cancer-related proteins, only serpinB5/maspin expression increased in PC9/ AZDR cells after AZD9291 treatment, suggesting a role in AZD9291 resistance (Fig 6A). To test whether maspin was involved in AZD9291 resistance, maspin expression was knocked down by siRNA in PC9/AZDR cells (Fig 6B). Downregulation of maspin reduced cell viability after AZD9291 treatment (Fig 6C).

## Discussion

The standard of care for patients with acquired resistance to EGFR-TKIs is rapidly changing after the development of third-generation EGFR TKIs targeting both EGFR T790M and activating EGFR mutations. AZD9291 (osimertinib) is the standard of care in patients with EGFR T790M-positive NSCLC after failure of prior EGFR TKI therapy [1, 15]. However, acquired resistance to AZD9291 has already been described in EGFR T790M-mutant NSCLC patients. The main mechanism of resistance to AZD9291 is the acquisition of an EGFR C797S mutation [8]. Additional mechanisms of resistance to AZD9291 include HER2 and MET amplification, RAS pathway activation, and MAPK activation [9–11, 14, 16]. AZD9291 has also showed promising anticancer activity in the first-line setting [17, 18]. Although it is anticipated that tumors will eventually develop resistance to AZD9291 in the first-line setting, the precise mechanism remains to be elucidated.

In this study, in a preclinical model of resistance to AZD9291 as first-line therapy, cells appeared to have bypassed EGFR signaling for survival. In the presence of AZD9291, they experienced sustained activation of downstream ERK signaling, despite decreased EGFR phosphorylation. Furthermore, phosphorylation of WNK1, a regulator of MAPK in EGFR signaling, was induced after AZD9291 treatment in AZD9291-resistant cells. WNK kinases are involved in the enhancement of cell proliferation and known as apoptosis inhibitors [19]. Thus increased activity of WNK1 caused by AZD9291 may be putative mechanism by which ERK activation is sustained in AZD9291-resistant cells.

The combination of MEK inhibitor with AZD9291 resensitized AZD9291-resistant cells. Our results are in line with previous reports that MEK inhibitors such as selumetinib (AZD6244) in combination with third-generation EGFR TKIs overcome acquired resistance [9, 10, 16, 20, 21].

Ras activation is an alternative bypass pathway of resistance in NSCLC. Resistance to EGFR TKIs may be related to increased dependency on RAS/MAPK signaling, including ERK activation [21]. A previous study demonstrated the efficacy of a combination of AZD9291 and the MEK inhibitor AZD6244 on the growth of NSCLC, regardless of EGFR T790M status [9]. Amplification of MAPK1 and NRAS Q61K mutations as well as copy-number gain of KRAS and NRAS were identified as mechanisms of resistance to AZD9291 in preclinical models [9]. In addition, the combination of a MEK inhibitor with AZD9291 restored the sensitivity of AZD9291-resistant cells, including those with MET amplification, an EGFR C797S mutation, or unknown mechanisms [16]. The authors showed that modulation of ERK-dependent Bim and Mcl-1 degradation are critical for anti-tumor activity in NSCLC harboring EGFR-activating mutations. Furthermore, reactivation of ERK signaling has also been reported in a drug resistance model to WZ4002 [20, 21], indicating that ERK signaling-mediated resistance is not AZD9291-specific. However, the reason why the MEK/ERK pathway in AZD9291 resistant cells is irresponsive to AZD9291 treatment has not been fully elucidated.

Our study also found an HRAS G13R mutation in PC9/AZDR cells. Although a previous case report identified an HRAS G13D mutation in the resistance to anti-EGFR monoclonal antibodies in colorectal cancer [22], this is the first report of an HRAS-activating mutation conferring acquired resistance to AZD9291 in NSCLC. HRAS mutations have been found in various cancer types and represent 1% of all mutations in NSCLC [23, 24]. An HRAS Q61L mutation in NSCLC might be aggressive and was associated with poor overall prognosis [25]. In a phase I trial, the MEK inhibitor RO5126766 induced 20% tumor shrinkage in patient with an HRAS mutation [26]. However, the molecular mechanisms behind drug resistance of HRAS mutations are poorly described. Though HRAS reduction has no effects on basal ERK phosphorylation levels in PC9/AZDR cells, the exact function of the HRAS G13R mutation should be tested.

In this study, we identified MET overexpression in AZD9291-resistant cells regardless of MET amplification. However, PC9/AZDR cells were not MET dependent, as MET inhibition was not sufficient to restore drug-sensitivity to AZD9291. Because AZD9291 reduced MET expression in AZD9291-sensitive cells, we speculated that AZD9291 could be involved in MET degradation. Thus, increased MET expression in resistant cells was not the cause of resistance but rather the result of acquired resistance. To verify this hypothesis, more studies need to be done.

To uncover additional resistance mechanisms, we analyzed expression of 84 cancer-related proteins using a proteome profiler array and found that maspin was elevated in PC9/AZDR cells after AZD9291 treatment. Maspin is a mammary serine protease inhibitor that is encoded by the *SERPINB5* gene and inhibits invasion and metastasis of cancer cells as a tumor suppressor [27]. Maspin inhibits cell motility by suppressing Rac1 and PAK1 activity and promotes cell adhesion via the PI3K/ERK pathway [28]. In addition, EGFR signaling promotes maspin phosphorylation and nuclear localization, where it inhibits gene transcription [29]. Although our results showed that resistance to AZD9291 partially arise through maspin, further study is needed to determine how maspin is involved in AZD9291 resistance.

## Conclusion

In summary, this study demonstrated a critical role of ERK activation in resistance to AZD9291 as a first-line therapy. The combination of AZD9291 and a MEK inhibitor may be an effective strategy to not only treat AZD9291-resistance in second-line therapy but also to treat AZD9291-resistance in first-line therapy.

## Supporting information

S1 Fig Sequencing result of EGFR T790M in PC9 and PC9/AZDR cells. EGFR T790M mutation was not found in PC9/AZDR cell.
(TIF)

S2 Fig Body weight of PC9/AZDR xenografts. PC9/AZDR xenografts were treated with vehicle, AZD9291 (5 mg/kg/d), AZD6244 (10 mg/kg/d), or AZD9291 plus AZD6244 by oral gavage for 5 days each week for a total of 4 weeks. Body weights were measured as indicated. Each measurement is mean ± SE of 9–10 replicates.
(TIF)

S3 Fig The combination of AZD9291 with MET inhibitors in PC9/AZDR cells. Cells were treated with various concentrations of AZD9291 alone, MET inhibitor (cabozantinib, capmatinib, MGCD-265, or Merestinib) alone, or their combinations for 72 h. The data are mean ± SE of six replicates.
(TIF)
