# Supplementary figures and images for: Acquired resistance to AZD9291 as an upfront treatment is dependent on ERK signaling in a preclinical model
Source: PLoS One. 2018 Apr 11;13(4):e0194730. doi: 10.1371/journal.pone.0194730 (PMC5895014; doi:10.1371/journal.pone.0194730)

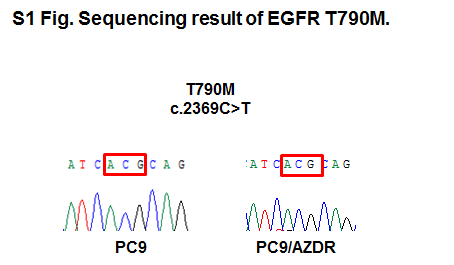

Supplement: S1 Fig — EGFR T790M mutation was not found in PC9/AZDR cell. (TIF) [file pone.0194730.s001.tif]

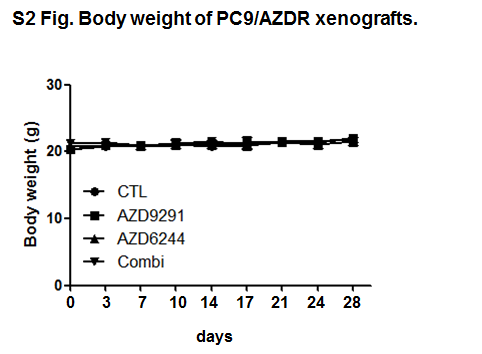

Supplement: S2 Fig — PC9/AZDR xenografts were treated with vehicle, AZD9291 (5 mg/kg/d), AZD6244 (10 mg/kg/d), or AZD9291 plus AZD6244 by oral gavage for 5 days each week for a total of 4 weeks. Body weights were measured as indicated. Each measurement is mean ± SE of 9–10 replicates. (TIF) [file pone.0194730.s002.tif]

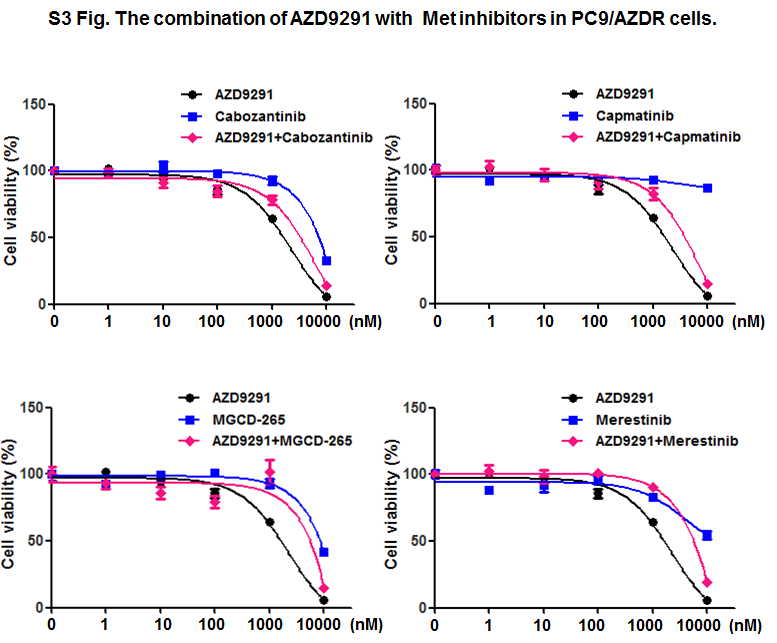

Supplement: S3 Fig — Cells were treated with various concentrations of AZD9291 alone, MET inhibitor (cabozantinib, capmatinib, MGCD-265, or Merestinib) alone, or their combinations for 72 h. The data are mean ± SE of six replicates. (TIF) [file pone.0194730.s003.tif]
